# Psychophysiological Stress Reactivity Relationships across the Menstrual Cycle

**DOI:** 10.1155/2015/631250

**Published:** 2015-12-16

**Authors:** Karen C. Olson, Haley A. Carroll, M. Kathleen B. Lustyk

**Affiliations:** 1 Lustyk Laboratory, Seattle Pacific University, 3307 3rd Avenue West, Seattle, WA 98119, USA; 2 Phoenix VA Health Care System, Topaz Clinic, 650 East Indian School Road, Phoenix, AZ 85012, USA; 3 University of Washington, NE 45th Street and 17th Avenue NE, Seattle, WA 98105, USA

## Abstract

While evidence suggests that women exhibit psychophysiological differences in stress reactivity across the menstrual cycle, the relationships among psychological and physiological stress reactivity states are not well understood. Healthy, normally cycling women (*N* = 44) participated in two counterbalanced laboratory sessions during the follicular and luteal phases where heart rate and subjective stress were assessed in response to stressors. There were no differences in the magnitudes of psychophysiological stress responses across the cycle. Psychological and physiological states were largely unrelated in the follicular phase but interrelationships were found in the luteal phase and these relationships were influenced by autonomic perception and trait anxiety. For women with high trait anxiety, autonomic perception appeared to buffer psychological and physiological stress reactivity during the luteal phase, suggesting that autonomic perception may be a protective factor for more anxious women during times of acute stress.

## 1. Introduction

While the stress response is adaptive [1], maladaptive reactivity has been linked to many health problems in women (e.g., coronary artery disease [2]). Evidence typically suggests that women experience higher levels of stress in the luteal phase compared to the follicular phase of the menstrual cycle such as discussed by Gordon and Girdler [3], yet little is known about the relationships among physiological and psychological measures of reactivity.

In recent years, adaptive forms of body awareness have become a greater focus of study. For instance, Lustyk and colleagues [4] found that women with lower levels of self-reported body awareness displayed higher hemodynamic reactivity in response to a stressor than did women with higher body awareness. However, there was no effect on subjective stress perhaps because body awareness encompasses a wide variety of sensations (e.g., body position).

Given these emerging findings, the present study investigated the relation between autonomic perception, or the subjective awareness specifically of physiological arousals (e.g., heart rate), and psychophysiological relationships across the menstrual cycle. We hypothesized that (1) women would exhibit greater psychophysiological responses to stressors during the luteal phase, (2) psychophysiological responses to stressors would be positively related, and (3) autonomic perception would moderate the relation between psychophysiological responses to stressors.

## 2. Materials and Methods

### 2.1. Power.

Power analyses were performed with phase comparison using the 2 (follicular × luteal) by 2 (baseline × stressor) MANOVA in G-Power [5]. We chose a conservative estimate by setting our effect size input parameters as small-moderate with *f*^2^ = .15, *α* = .05, and 1–*β* = .80 and relatively large correlations among the repeated measures [6]. Given 40 participants, the result of the power analysis was .8041.

### 2.2. Participants.

Participants were recruited via advertisements in Seattle, Washington, after obtaining Institutional Review Board approval. Interested participants were screened over the phone. Since upward of 10% of cycles may be anovulatory, which adds to the risk for dropout [7], we incorporated planned missingness strategies into our study [8]. These strategies included pulsing our advertising throughout the study and screening continuously until our projected sample size was met. All participants indicated that they had not been diagnosed with physical or mental illness and reported that they were nonsmokers and they were not daily drinkers. Women who reported use of oral contraceptives [9, 10], chronic health conditions such as hypertension [11], and use of medications affecting the stress response such as xanthine drugs [12] were not included in the study. Our resultant sample included 44 females. Participants were mainly Caucasian (*n* = 34) and between 18 and 30 years old (*n* = 31).

### 2.3. Psychophysiological Measures of Stress.

Heart rate (HR) was obtained with a 3-lead electrocardiography (ECG) and continuously monitored throughout the procedure via the PowerLab data acquisition system (PowerLab 800; AD Instruments, Boulder, CO).

### 2.4. Psychological Measures of Stress.

The State-Trait Anxiety Inventory (STAI) was chosen in accordance with prior research indicating that it reliably captures subjective stress while minimally interfering with laboratory procedures [13, 14]. The questionnaire has two scales with 20 items each assessing state (STAI-S) and trait (STAI-T) anxiety. Participants rate statements such as “I am calm” on a Likert scale indicating “how you feel right now, i.e., at this moment” for STAI-S and “I am a steady person” for STAI-T from 0 (*not at all*) to 4 (*very much so*). Scores were reverse coded such that higher scores reflect anxiety. Cronbach’s alpha STAI-S = .92 and STAI-T = .91.

### 2.5. Autonomic Perception.

The 90-item Autonomic Perception Questionnaire-Revised (APQ-R) measures perception of physiological arousal during states of anxiety, anger, and sadness [15, 16]. Participants rate each item (e.g., “my face becomes hot”) on a Likert scale indicating “when I feel… (anxious/angry/sad)” from 1 (*not at all true about me*) to 9 (*very true about me*), with 5 indicating (*neutral, not sure*). The APQ-R is summed within three 30-item subscales of anxiety, anger, and sadness. Higher scores indicate more perceptions of autonomic arousals. Cronbach’s alpha anxiety = .93, anger = .94, and sadness = .94.

### 2.6. Laboratory Stressors.

The Paced Auditory Serial Addition Test (PASAT) and the cold pressor task were chosen in accordance with prior research suggesting that cognitive and physical stressors elicit similar response in women irrespective of cycle phase. The PASAT is a cognitive stressor known for evincing negative affect and a physiological stress response [14, 17]. Participants audibly added pairs of numbers at a progressively rapid pace. The cold pressor is a physical stressor shown to produce measurable psychological and hemodynamic reactivity [14, 18]. Participants placed their hand in warm water (35–37°C) for 4 minutes and then transferred their hand to cold water (1–3°C) for as long as they could tolerate it (2 minutes maximum) and then into the warm water for another 4 minutes.

### 2.7. Procedure.

Participants completed two laboratory-testing sessions during the course of one menstrual cycle (follicular days 5–9 where start of menses was day one and luteal days 7–9 where day of ovulation was day one). Ovulation was confirmed with a take-home urine test, which detects the luteinizing hormone (LH) surge with 98% accuracy (Answer Quick: Scantibodies Laboratory, Inc., Santee, CA). Stressor task was randomized by cycle phase to counter habituation effects. Participants abstained from alcohol, tobacco, and over-the-counter medications within 24 hours of the session, engaging in heavy exercise the morning of the session, and food and caffeine within one hour of the session. All sessions were conducted between 10:00 am and 4:00 pm to avoid stress testing during marked changes in the diurnal cortisol slope [19].

At the start of testing, participants provided written and oral consent after discussion of the procedure with the examiners. Physiological measures were obtained during a 15-minute *baseline* where the participant listened to relaxing music and completed the STAI-S. Following baseline, the *stressor* phase began where participants performed either the PASAT or cold pressor task per randomization. Immediately after the stressor, participants completed a second STAI-S and then relaxed for a 15-minute *recovery* period. After the test session, each participant was given a take-home packet including the STAI-T and APQ-R. A more complete laboratory procedure has been published [14].

## 3. Results

Data reduction for HR resulted in means for the 15 min baseline and stressor period (see Table 1). Reactivity change scores were calculated by subtracting the mean baseline score from the mean stress score. The main effect of stressor type was not statistically significant for HR (Wilk’s Λ = .75, *F*(2, 21) = 3.56, *p* = .05, multivariate *η*^2^ = .25) or STAI (Wilk’s Λ = .77, *F*(5, 37) = 2.24, *p* = .07, multivariate *η*^2^ = .23). Thus, physiological variables for the PASAT and cold pressor were collapsed. No reductions were applied to state anxiety data. Between cycle phase, paired-samples *t*-tests revealed that the degree of autonomic perception within follicular phase, anxious (*M* = 148.24, SD = 37.34), angry (*M* = 143.00, SD = 39.65), and sad (*M* = 123.81, SD = 41.09), were not significantly different from luteal phase, anxious (*M* = 150.64, SD = 37.56, *t*(41) = −.55, *p* = .59, *R*^2^ = .50), angry (*M* = 142.23, SD = 38.70, *t*(41) = .18, *p* = .86, *R*^2^ = .58), and sad (*M* = 126.57, SD = 44.82, *t*(41) = −.74, *p* = .46, *R*^2^ = .72). These scales were collapsed into one scale of their total scores averaged together.

### 3.1. Psychophysiological Reactivity Across Menstrual Cycle.

First, a 2 (baseline × stressor) by 2 (follicular × luteal) MANOVA assessed physiological reactivity to the stressor between the follicular and luteal phase. There was a main effect of time as indicated by higher HR during the stressor period (Wilk’s Λ = .29, *F*(1, 41) = 101.45, *p* < .001, multivariate *η*^2^ = .71).The interaction revealed that heart rate reactivity was greater during the luteal stressor period (Wilk’s Λ = .83, *F*(1, 41) = 8.24, *p* < .01, multivariate *η*^2^ = .17). However, paired-samples *t*-tests revealed that the magnitude of reactivity in HR as calculated by the mean baseline score subtracted from the mean stressor score was similar across the menstrual cycle (*t*(42) = −.38, *p* = .71, *R*^2^ = .07).

Additionally, MANOVA assessed psychological reactivity to the stressor between the follicular and luteal phase. There was a main effect of time as indicated by higher STAI scores during the stressor period (Wilk’s Λ = .10, *F*(5, 37) = 69.3, *p* < .001, multivariate *η*^2^ = .90) but with no interaction (Wilk’s Λ = .77, *F*(5, 37) = 2.24, *p* = .07, multivariate *η*^2^ = .23). Again, there were no differences in the magnitude of reactivity as calculated by change scores across the menstrual cycle (*t*(42) = −.20, *p* = .84, *R*^2^ = .01).

Next, bivariate correlations assessed the relation between physiological and psychological reactivity states across the menstrual cycle. During the follicular phase, psychological and physiological states were largely unrelated (see Table 2). During the luteal phase, baseline HR was related to stress response STAI-S (*r* = .35) and STAI-T (*r* = −.37; *p* < .05).

### 3.2. Moderation of Psychophysiological Reactivity by Autonomic Perception.

**Figure 1:**
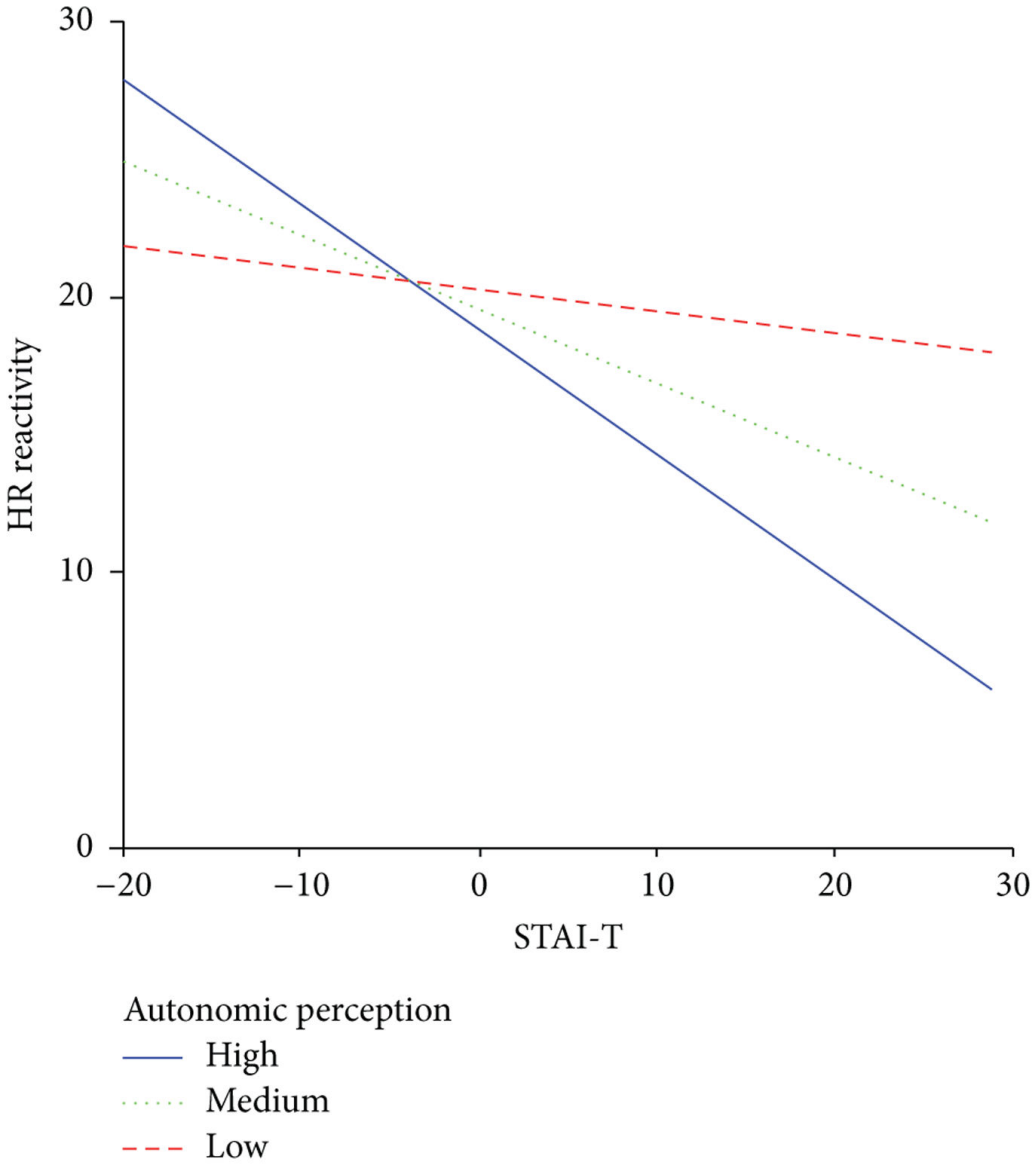
Moderation of luteal heart rate reactivity predicted by mean centered trait anxiety by autonomic perception. Note: HR = heart rate in beats per minute; reactivity = baseline value subtracted from stress response value; STAI-T = trait portion of the State-Trait Anxiety Questionnaire; autonomic perception = Autonomic Perception Questionnaire-Revised.

Finally, multiple regression results revealed that the relation between psychological (follicular *F*(4, 36) = 1.66, *p* = .18, *R*^2^ = .14) and physiological reactivity (follicular *F*(4, 36) = 1.46, *p* = .23, *R*^2^ = .16) was moderated by autonomic perception during the luteal phase only. The interaction between the STAI-T and the APQ-R indicated that, as STAI-T scores increased, participants with higher APQ-R scores had lower STAI-S stress response scores than participants with lower APQ-R scores, *R*^2^ change = .21, *F*(3, 40) = 3.52, *p* < .05. In addition, the interaction indicated that, as STAI-T scores increased, participants with higher APQ-R scores had lower HR reactivity than did participants with lower APQ-R scores, adjusted *R*^2^ = .11, *F*(3, 40) = 2.73, *p* < .05 (see Figure 1).

## 4. Discussion

Our first hypothesis that there would be greater psychophysiological responses to stressors during the luteal phase was not supported. While there were marked increases in both physical and psychological markers to the stressor, there were no differences in the magnitude of reactivity across the cycle as calculated by change scores. This finding is inconsistent with prior literature [14] but appears to be driven by higher baseline levels of psychophysiological states during the luteal phase when compared to the follicular phase.

Our second and third hypotheses that psychophysiological variables would be positively related and that autonomic perception would moderate this relationship were partially supported. Interestingly, relationships were not found among psychophysiological states in the follicular phase despite simultaneous reactivity to the stressors being observed. However, in the luteal phase, HR was associated with the luteal subjective stress response and with trait anxiety. Similarly, there appeared to be no effect of autonomic perception in the follicular phase but, in the luteal phase, the relationship between the luteal subjective stress response and HR reactivity was buffered by higher levels of autonomic perception. This is consistent with other findings that individual characteristics, such as neuroticism, can lead to more exaggerated stress responding [20] and that mindful body movements can lower stress reactivity [21]. As therapies using a form of autonomic perception, mindfulness (e.g., Mindfulness Based Stress Reduction), [22] emerge, it is important to further understand the relation between perception of bodily sensations and stress reactivity.

Some limitations of the study include that although there were no differences in stressor performance or in magnitude of stress response across the menstrual cycle, some of the effects from the luteal phase could be due to learning. The present study design restricted the characteristics of the participants and, in doing so, excluded many women who have relatively common medical or mental health conditions. A small sample size may have been one of the reasons why psychophysiological relationships were generally not found in the follicular phase. Also, these results may not generalize well to women with clinical diagnoses of anxiety where focus on autonomic perceptions without relaxation training could potentially exacerbate symptoms.

## 5. Conclusions

Important psychophysiological differences in stress reactivity across themenstrual cycle continue to be found. There may be a buffering effect of autonomic perception on stress reactivity in the luteal phase. For women with higher trait anxiety, there was evidence to suggest that women with greater awareness of their body states had less psychological and physiological reactivity during times of acute stress. Given well established relationships between stress and health problems in women, these findings may have important implications for detection, prevention, and treatment of disease.

## Figures and Tables

**Table 1:** Descriptive values for psychological and physiological baseline, stress response, and reactivity across the menstrual cycle phase (*N* = 44).

	Follicular		Luteal	
	M	SD	M	SD
STAI-S				
Baseline	30.57	7.38	30.84	9.16
Stress response	54.71	8.78	54.02	11.03
Reactivity	24.14	9.42	23.18	10.63
HR				
Baseline	62.82	8.35	66.41	10.44
Stress response	71.11	9.85	75.27	11.31
Reactivity	8.22	6.55	8.86	7.16

*Note*. STAI-S = state portion of the State-Trait Anxiety Questionnaire; HR = heart rate in beats per minute; baseline = 15-minute mark during the first fifteen minutes; stress response = mean of stressor task period; reactivity = baseline value subtracted from stress response value; M = mean; SD = standard deviation.

**Table 2:** Bivariate correlations between psychological and physiological states during baseline, stress response, and reactivity.

		Follicular STAI-S		STAI-T
	Baseline	Stress response	Reactivity Δ	Trait anxiety
Follicular HR				
Baseline	.05	.23	.17	.25
Stress response	−.13	.15	.24	.04
Reactivity	−.30	.10	.33*	−.19
		Luteal STAI-S		STAI-T
	Baseline	Stress response	Reactivity Δ	Trait anxiety
Luteal HR				
Baseline	.10	.35*	.09	.06
Stress response	.06	.17	.13	.06
Reactivity	−.16	.08	.06	−.37*

*Note*. HR = heart rate in beats per minute; STAI-S = state portion of the State-Trait Anxiety Questionnaire; STAI-T = trait portion of the State-Trait Anxiety Questionnaire; baseline = mean of baseline period; stress response = mean of stressor task period; reactivity = baseline value subtracted from stress response value.

* *p* < .05.

## Highlights

Higher luteal baseline states resulted in higher stress response states. Psychophysiological relationships were found during the luteal phase only. Luteal subjective stress and heart rate reactivity were buffered by autonomic perception.
